# *UPDhmm*: detecting uniparental disomy from NGS trio data

**DOI:** 10.1093/bioinformatics/btag062

**Published:** 2026-03-13

**Authors:** Marta Sevilla-Porras, Carlos Ruiz-Arenas, Luis A Pérez-Jurado

**Affiliations:** Department of Medicine and Life Sciences, Universitat Pompeu Fabra, Barcelona, 08003, Spain; Center for Biomedical Network Research on Rare Diseases (CIBERER), Instituto de Salud Carlos III, Madrid, 28029, Spain; CIMA—University of Navarra, Pamplona, 31008, Spain; Institute of Health Research of Navarra (IdiSNA), Pamplona, 31008, Spain; Department of Medicine and Life Sciences, Universitat Pompeu Fabra, Barcelona, 08003, Spain; Center for Biomedical Network Research on Rare Diseases (CIBERER), Instituto de Salud Carlos III, Madrid, 28029, Spain; Genetics Service, Hospital del Mar and Hospital del Mar Research Institute, Barcelona, 08003, Spain

## Abstract

**Summary:**

Uniparental disomies (UPDs) are copy-neutral chromosomal alterations that occur when both copies of a chromosome pair (entire or segmental) come from one parent. UPDs, including isodisomies (identical parental chromosome) and heterodisomies (two different homologs from the same parent), reflect meiotic and/or mitotic aberrations of chromosomal segregation that can be associated with congenital or acquired disease. Despite their relevance, current methods to detect UPDs using sequence data (exomes or genomes) have limited sensitivity for small events, cannot precisely determine the UPD sub-type or coordinates, and perform poorly when including individuals or populations with consanguinity. We present *UPDhmm*, a novel tool that uses trio-based sequence data (proband and parents) and models inheritance patterns. *UPDhmm* predicts the most likely inheritance scenario, normal Mendelian inheritance versus UPD event, based on genotype combinations using a Hidden Markov Model (HMM). We validated the method using simulations on exome and genome data from 1000-Genomes projects. *UPDhmm* overperformed currently available methods in detecting simulated UPD events in both data types. We applied *UPDhmm* to a collection of nearly 2400 families with a proband with autism spectrum disorder (Simons Simplex Collection Project) and identified UPD events in two affected individuals, one of them previously unreported. These two events, a paternal isodisomy of chr8 and a maternal heterodisomy of chr22, can be genetic causes of the disease, demonstrating the clinical utility of *UPDhmm*. Thus, *UPDhmm* can facilitate the incorporation of UPD detection into clinical pipelines of genomic analysis.

**Availability and implementation:**

*UPDhmm* is implemented in R and is available in the Bioconductor package (version 1.5.0): https://www.bioconductor.org/packages/release/bioc/html/UPDhmm.html. The source code can be found at https://github.com/martasevilla/UPDhmm under the MIT license.

## 1 Introduction

Uniparental disomies (UPDs) occur when the two homologous chromosomes of an individual (or a segment of those chromosomes) come from a single parent. They include isodisomies, where the two homologs derive from the same parental chromosome, heterodisomies, where they derive from the two different homologs of one parent, and mixed, with alternating stretches of isodisomy and heterodisomy (Shaffer *et al.* 2001). UPDs can be maternal or paternal, depending on the parental origin of the chromosome pairs, and can affect single chromosomes, chromosomal segments, or the entire genome. Whole chromosome UPDs are thought to mainly originate from postzygotic rescue of meiotically derived zygotic trisomy (resulting in heterodisomy or mixed UPD) or monosomy (resulting in isodisomy) following chromosomal non-disjunction. These corrections can restore disomic balance but may also generate isodisomy or heterodisomy (depending on meiotic recombination) and sometimes result in residual tissue-specific mosaicism, that can cause phenotype in the tissues where the monosomy or trisomy is still present. Segmental UPDs typically arise from interchromatid exchange by somatic repair of DNA double-strand breaks (Mackay *et al*. 2018, Papenhausen *et al.* 2021).

UPDs can contribute to disease either as causative or protective mechanisms. As a causative factor, they can lead to disease through homozygosity of pathogenic recessive mutations or errors in genomic imprinting. On the other hand, UPDs may act as a protective mechanism by rescuing lethal or pathogenic dominant mutations, thereby allowing the embryo or the tissue to survive otherwise incompatible genomic alterations (Liehr 2022, Moch *et al.* 2024). The prevalence of detectable UPDs is much higher in patients with suspected genetic conditions (ranging from 1 in 1400 to 1 in 200) (Yauy *et al.* 2020, Scuffins *et al.* 2021, Wang *et al.* 2021) compared to the estimated prevalence in the general population (1 in 2000 births) (Nakka *et al.* 2019) showing its biological impact. Segmental UPDs increase with aging and are common in cancer and cancer-predisposing disorders like Fanconi anemia (Jacobs *et al.* 2012, Reina-Castillón *et al.* 2017). Despite their role in disease and the proposed guidelines of the American College of Medical Genetics for diagnostic testing (del Gaudio *et al.* 2020), UPDs are not commonly evaluated during routine genetic analysis so they are missed.

UPD detection relies on identifying an allele combination in the proband incompatible with the genotypes of one of the parents. UPDs can be detected using low scale experimental approaches or using computational methods and genome-wide data. The gold standard for confirming UPD events is Short Tandem Repeat markers (STRs), an experimental method that involves genotyping polymorphic loci in the proband and parents to assess the inheritance pattern (Shaffer *et al.* 2001, del Gaudio *et al.* 2020). Computational methods rely on two main approaches to identify UPD events from genome-wide genetic data. One approach detects runs of homozygosity (ROH, i.e. long stretches of homozygous variants), indicative of isodisomy, as implemented in *AltAFplotter* (Radtke *et al.* 2024). The second approach focuses on analyzing trio (proband, father, mother) or duo (proband, parent) datasets by identifying informative genomic positions—i.e. genetic loci where the inheritance pattern is only compatible with Mendelian inheritance or results from UPD. Then, UPD events are identified as regions with a high proportion of positions exclusive of UPD inheritance, as implemented in *UPDio* (King *et al.* 2014) and *AltAFplotter* (Radtke *et al.* 2024). However, these methods have not been evaluated in whole-genome sequencing (WGS) data and are not well-suited for samples from consanguineous individuals. Additionally, these methods cannot determine the coordinates of the UPD event.

To address the limitations of existing methods for UPD detection from sequence data, we have developed *UPDhmm*, a novel R/Bioconductor package. *UPDhmm* models Mendelian and UPD inheritance patterns to accurately detect both isodisomies and heterodisomies. *UPDhmm* is designed for trio-based datasets and can identify the genomic coordinates of UPD events enabling high-throughput detection on next-generation sequencing (NGS) data. We evaluated *UPDhmm* on a cohort of autism families, the Simons Simplex Collection (SSC). In this context, *UPDhmm* identified two relevant UPD events, demonstrating its clinical applicability.

## 2 Materials and methods

### 2.1 UPDhmm implementation

**Figure 1:**
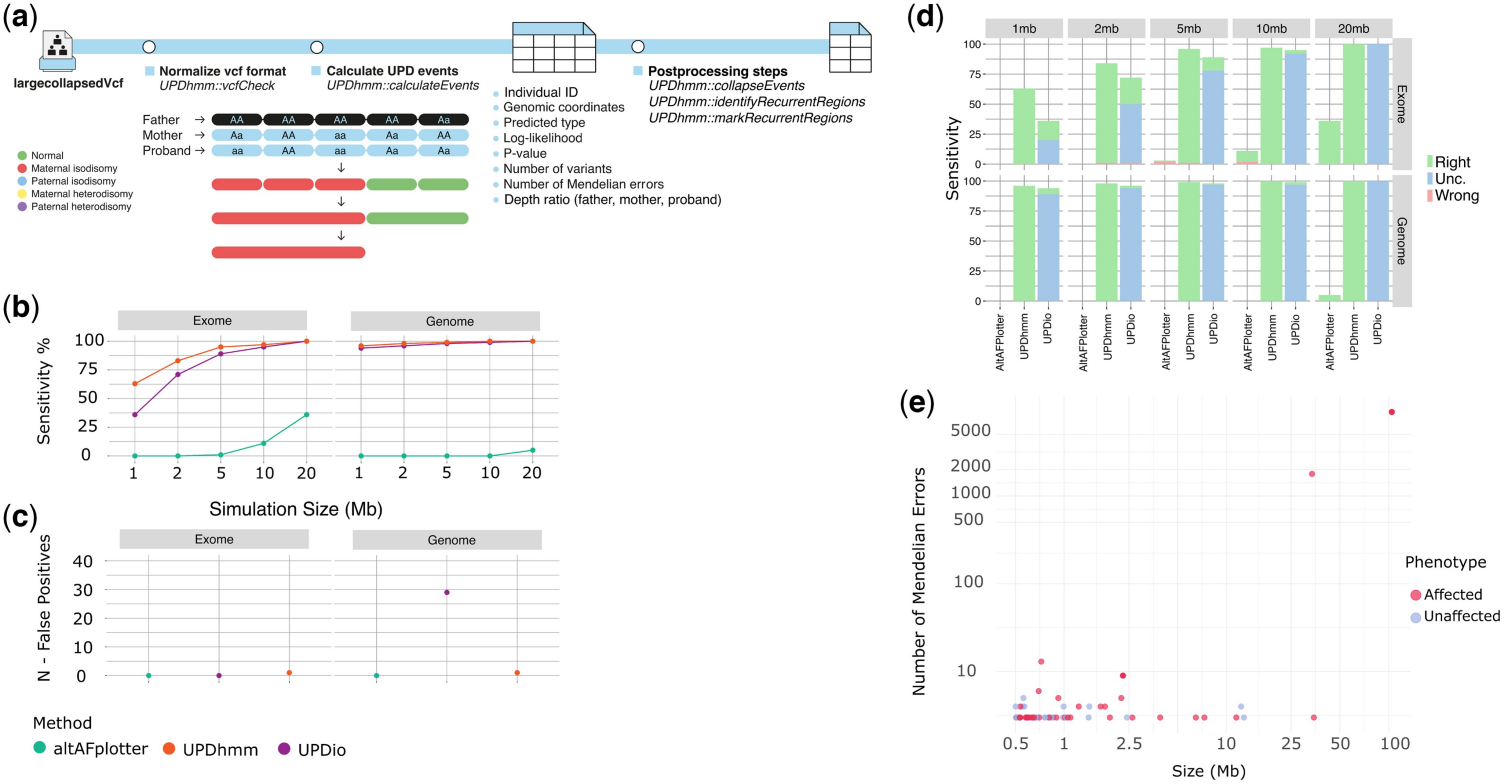
Evaluation of *UPDhmm* performance in simulated datasets and its application to autism cohort data. (a) Workflow of the *UPDhmm* package. *UPDhmm* consists of two main functions: (i) vcfCheck and (ii) calculateEvents. Postprocessing steps can be performed with native functions: collapseEvents(), identifyRecurrentRegions() and markRecurrentRegions(). calculateEvents() function produces a data.frame with annotated events, including chromosome, start, end, type, log-likelihood, *P*-value, the number of SNPs within the regions, the number of Mendelian errors, Individual ID, and depth ratio of the three individuals of the trio. (b and c) Benchmark evaluation of *UPDhmm*, *UPDio*, and *AltAFplotter* on simulated UPD events. (b) Detected simulated events by event sizes. (c) False positive events, defined as detected events other than the simulated UPD events. (d) Classification of simulated UPD events across different methods. For each method, the number of detected events is categorized based on classification quality: correctly classified (*Right*), incorrectly classified (*Wrong*), and uncertain classification, when both types are detected (*Unc.*) across different simulation sizes in both exome (upper panel) and genome (lower panel) data. (e) Distribution of UPD events in the SSC cohort after the filtering strategy by estimated size of the event (*x*-axis) and number of Mendelian errors (*y*-axis). Dots are colored based on the individuals’ phenotype.

*UPDhmm* utilizes a Hidden Markov Model (HMM) to represent inheritance patterns observed during normal meiosis (Mendelian inheritance) or in UPD events, such as maternal isodisomy, paternal isodisomy, maternal heterodisomy, and paternal heterodisomy (Supplementary Text, available as supplementary data at *Bioinformatics* online). The package accepts a VCF-class object based on the RangedSummarizedExperiment class of Bioconductor. The workflow uses two core functions: **vcfCheck()** and **calculateEvents()** (Fig. 1a). The **vcfCheck()** function pre-processes the input, ensuring the VCF-class object is properly formatted for *UPDhmm* and can optionally run a quality control check based on Genotype Quality (>20) and Read Depths (>30). The **calculateEvents()** function identifies UPD events in four key steps: (i) splitting the VCF by chromosome; (ii) applying the Viterbi algorithm to each chromosome to determine the most likely sequence of hidden states (i.e. inheritance patterns) for each variant; (iii) aggregating contiguous variants with the same state into larger genomic segments; (iv) computing the log-likelihood ratio and *P*-values of the comparison between UPD inheritance and normal inheritance (iv) and calculating the depth ratio between the detected event and the rest of the genome for every individual of the trio. The final output is a comprehensive table that includes individual ID, genomic coordinates (chromosome, start, and end positions), predicted UPD type (e.g. paternal isodisomy), the number of Mendelian errors for each predicted block, the log-likelihood ratio and *P*-value and depth ratio of the proband, father and mother.

Additionally, UPDhmm provides several post-processing functions to refine and interpret UPD results. The collapsedEvents() function merges UPD calls of the same type and chromosome into a single event, while identifyRecurrentRegions() and markRecurrentRegions() allow users to detect and annotate recurrent regions across samples.

### 2.2 Benchmark evaluation

Simulated UPD events were introduced in two exome datasets and ten genome datasets from the 1000 Genomes Project (Table 1, available as supplementary data at *Bioinformatics* online). Pre-processing steps were conducted to remove conflicting regions (Supplementary Text, available as supplementary data at *Bioinformatics* online).

We assessed *UPDhmm*, *UPDio*, and *AltAFplotter* by measuring sensitivity, false positives, and UPD type classification. For *UPDhmm* and *UPDio*, detection required matching the UPD type (iso/hetero), parental origin (paternal/maternal), and chromosome. For *AltAFplotter*, detection was based on the criteria proposed in the original study (Moch *et al.* 2024) (Supplementary Text, available as supplementary data at *Bioinformatics* online). False positives were defined as any events different from simulated UPDs. As methods can assign multiple UPD types to the same UPD event, we categorized detected UPD events into right, wrong and uncertain. (Supplementary Text, available as supplementary data at *Bioinformatics* online). Additionally, we evaluated *UPDhmm’*s accuracy in identifying the coordinates of simulated UPD events. We also explored the impact of structural variants (SVs) on the methods’ performance. False positives were compared before and after filtering structural variants (Supplementary Text, available as supplementary data at *Bioinformatics* online).

### 2.3 UPDhmm evaluation using real data: autism spectrum disorder (ASD) dataset

We assessed *UPDhmm’*s performance in identifying UPD events in the Simons Simplex Collection (SSC), an autism cohort. We analysed blood-derived genotype WGS data from 9065 individuals across 2379 families. SSC cohort encompassed 2379 probands diagnosed with autism and 1928 unaffected siblings, organized into 451 trios and 1928 quads (Supplementary Text, available as supplementary data at *Bioinformatics* online).

Before applying *UPDhmm*, samples were pre-processed to assess quality and remove conflicting regions (Supplementary Text, available as supplementary data at *Bioinformatics* online).

## 3 Results

### 3.1 *UPDhmm* implementation

*UPDhmm*, implemented as an R/Bioconductor package, identifies uniparental disomy (UPD) events using genomic data from trio datasets. *UPDhmm* relies on selecting chromosomal regions with non-Mendelian inheritance patterns.

### 3.2 *UPDhmm* outperforms other methods in simulated data

We compared the performance of *UPDhmm* against *UPDio* and *AltAFplotter* using simulated UPD events from exome and genome data derived from the 1000 Genomes Project. Optimal thresholds for *UPDio* and *UPDhmm* were established using simulated data, as detailed in the Supplementary Methods (Figs 1 and 2, available as supplementary data at *Bioinformatics* online). *AltAFplotter* uses hard-coded parameters for tagging chromosomes.

*UPDhmm* outperformed *UPDio* and *AltAFplotter* in simulated UPDs. *UPDhmm* had higher sensitivity than *UPDio* in detecting smaller UPD events (up to 2 Mb) in both exome and genome data, while both achieved a 100% sensitivity for larger events (≥5 Mb). *AltAFplotter* exhibited low performance in both exome and genome simulations due to incorrectly flagging most individuals as consanguineous, preventing proper UPD evaluation (Fig. 1b). Regarding false positive detection, *UPDhmm* demonstrated a lower number of false positives compared to *UPDio*, while *AltAFplotter* did not detect false positive events (Fig. 1c). Removing regions with SVs reduced false positives in both *UPDhmm* and *UPDio* (Fig. 3, available as supplementary data at *Bioinformatics* online). Furthermore, while no major performance differences between maternal and paternal UPD, both methods had a lower performance detecting heterodisomies smaller than 10 Mb in exome data (Fig. 4, available as supplementary data at *Bioinformatics* online).

*UPDhmm* accurately differentiated between heterodisomies and isodisomies (Fig. 1d), while *UPDio* classified most events as both isodisomies and heterodisomies. Furthermore, *UPDhmm* provided precise chromosomal coordinates. Overall, over 80% of the simulated events demonstrated an overlap of 90%–100% with their corresponding simulated coordinates (Fig. 5, available as supplementary data at *Bioinformatics* online).

### 3.3 Detection of UPDs in ASD patients with UPDhmm

We applied *UPDhmm* to WGS data from the Simons Simplex Collection (SSC), comprising 4307 trios (2379 ASD probands, 1928 siblings). Initially, 387 candidate UPD events were identified across 371 individuals (Table 2, available as supplementary data at *Bioinformatics* online). After excluding low-confidence calls (recurrent events) and potential copy number variants (CNVs) based on sequencing depth deviations, a final set of 69 UPD events in 67 individuals was retained (Fig. 1e) (Supplementary Text, available as supplementary data at *Bioinformatics* online).

From this set, we prioritized two large UPD events, both exceeding 50 Mb in size and compatible with whole chromosome UPD, with a high number of Mendelian errors. None of these events were due to CNVs, further validated with SNP array data (Fig. 6, available as supplementary data at *Bioinformatics* online). These UPDs were identified in unrelated ASD cases (SSC07047 and SSC11827). Patient SSC07047 presented a paternal isodisomy of chromosome 8, while patient SSC11827 showed a heterodisomy involving the entire chromosome 22. Patient SSC07047’s isodisomy was previously identified based on high rates of Mendelian errors (Trost *et al.* 2022) while patient SSC11827’s heterodisomy, to our knowledge, has not been previously detected.

## 4 Discussion

Uniparental disomies (UPDs) are chromosomal anomalies that can contribute to genetic diseases. To improve UPD detection, we developed *UPDhmm*, an R package designed to identify UPD events by modelling UPD inheritance patterns in trio-based datasets.

*UPDhmm* outperforms existing methods like *UPDio* and *AltAFplotter* in detecting uniparental disomy (UPD) events. *UPDhmm* relies on a HMM (Hidden Markov Model) probabilistic framework which leverages all available genomic variants, unlike previous methods which focus only on informative genomic variants (i.e. those exclusive to either Mendelian inheritance or UPD inheritance). The HMM model of *UPDhmm* also enhances the differentiation between isodisomies and heterodisomies. Additionally, *UPDhmm* avoids using runs of homozygosity, reducing confounding factors like consanguinity, and provides precise genomic coordinates, facilitating the connection between genetic variants and clinical phenotypes.

In our analysis of the SSC cohort, we identified two UPD events that could potentially explain the probands’ phenotypes: a chromosome 8 isodisomy and a chromosome 22 heterodisomy. The chromosome 8 isodisomy was previously interpreted as a diagnostic finding in a large-scale analysis focused on ASD-related genetic variants (Trost *et al.* 2022) despite no candidate homozygous pathogenic variants were reported within this region. A similar chromosome 22 heterodisomy has recently been described in an individual with comparable clinical features (Radtke *et al.* 2024), while chromosome 22 UPDs was associated with ASD in a population-based study (Nakka *et al.* 2019). In both cases, UPDs might not be the cause of the phenotype, but a consequence of aneuploidy rescue events. Thus, UPDs in blood might indicate the presence of monosomies or trisomies in other tissues of interest, which will be the real cause of the phenotype. Therefore, the events identified should be interpreted as candidate pathogenic mechanisms rather than diagnoses, warranting further functional or genetic analyses in other tissues.

Although UPDhmm initially reported a high number of UPD events in the SSC cohort, we proposed a set of filtering steps that increased the specificity of the method, enhancing robustness and interpretability. Notably, true UPD events often show a correlation between genomic size and the number of Mendelian errors. In contrast, events with few errors but large size typically map to poorly captured regions—such as centromeres—or reflect homozygosity from consanguinity.

A limitation of our work is the lack of a golden standard, i.e. datasets with validated UPD events, to evaluate UPD detection algorithms. Nonetheless, introducing UPD events into real genome and exome data will mostly recapitulate the features of true UPD events.

In conclusion, *UPDhmm* can be easily incorporated into the analysis or re-analysis of undiagnosed rare disease patients, given that it works with exome and genome data. Thus, *UPDhmm* offers a scalable strategy to improve genetic diagnosis.

## Supplementary Material

btag062_Supplementary_Data

## Data Availability

The data used in this paper is available at the 1000 Genomes Project sample collection. Genome data and exome data from Byrska-Bishop et al., 2022 and Sudmant et al., 2015, respectively and SSC Approved researchers can obtain the SSC population dataset described in this study (https://www.sfari.org/resource/simons-simplex-collection/) by applying at https://www.sfari.org/resource/sfari-base/.

## Code availability

The source code used for the analysis of simulated UPD events and the SSC cohort is available at https://github.com/martasevilla/UPDhmmData. The source code of the UPDhmm R package is available at https://github.com/martasevilla/UPDhmm.
