# Supplementary material for: UPDhmm: detecting uniparental disomy from NGS trio data
Source: Bioinformatics. 2026 Mar 13;42(3):btag062. doi: 10.1093/bioinformatics/btag062 (PMC13032891; doi:10.1093/bioinformatics/btag062)
Supplement: btag062_Supplementary_Data [file btag062_supplementary_data.zip › Supplementary_material.pdf]

Supplementary text

UPDhmm implementation

In our model, hidden states correspond to five inheritance patterns: normal inheritance (Mendelian), maternal isodisomy, paternal isodisomy, maternal heterodisomy and paternal heterodisomy. Emission probabilities are calculated based on the formula P(combination) = P(G_offspring) × P(G_mother) × P(G_father). We assume Hardy-Weinberg equilibrium (HWE) for parents' genotype frequencies, with equal allele frequencies for the reference and alternative alleles ($p=q=0.5$). Consequently, the genotype frequencies are 0.25 for homozygous genotypes and 0.5 for heterozygous genotypes. Genotype frequencies in the offspring depend on the paternal genotypes and the inheritance pattern. For normal inheritance, Mendelian rules apply. In the case of UPD inheritance, it depends on the genotype of the parent from whom the event originates. For isodisomy, if the parent is heterozygous, the offspring will be homozygous for either allele of the parent's genotype in equal proportions. If the parent is homozygous, the offspring will inherit the same homozygous genotype. For heterodisomy, the offspring's genotype matches the parent’s genotype. To ensure numerical stability within the emission probability matrix, some invalid genotype combinations under a given inheritance model are set to 1e-05. This probability is comparable to that of sequencing errors or de novo mutations (Ma *et al.* 2019). Additionally, a null observation is included to normalize all possible observations, ensuring that all the emission probabilities from

each state sum to 1. Start probabilities are set to favour the normal state, while transition probabilities are biased towards remaining in the same state. Detailed emission probabilities, transitions probabilities and start probabilities can be found in Supplementary Table 3.

Benchmark Evaluation

UPD events simulation creation

For isodisomy simulations, proband genotypes in a region were set as homozygous for the alleles of one parent. In contrast, for heterodisomy simulations, proband genotypes were defined as the exact genotype of one parent. We simulated events of 1, 2, 5, 10, and 20 megabases, conducting 100 simulations for each size in both exome and genome data. Regions for simulations were randomly created for each size, ensuring a minimum number of variants within each region (Supplementary Table 4).

AltAFplotter required criteria for UPD detection.

For *AltAFplotter*, detection was based on the criteria proposed in the original study: (1) isodisomies were defined as chromosomes tagged as ROH high or mixed; (2) heterodisomies were defined as chromosomes tagged as ROH high or mixed and with a high inheritance ratio tag; and (3) samples with chromosomes tagged as likely consanguineous were considered negative for UPD.

Parameters optimization on *UPDhmm* and *UPDio*

For *UPDhmm*, we excluded events with fewer than two Mendelian errors and assessed different event size thresholds: 100, 250, 500, and 1,000 Kb. For *UPDio*, we tested various -log10(p-value) thresholds: 2, 7.45, 10, 20, 30 being 7.45 the standard value from the method's initial publication(King *et al.* 2014). No parameter adjustments were made for *AltAFplotter*, as its cut-offs were predefined and fixed. For *UPDio*, we tested a range of p-value thresholds and selected the values that maximized the sensitivity-specificity trade-off. The optimal -log10(p-value) threshold was 7.45 for exome datasets and 20 for genome datasets (see Figures S1–S2). In contrast, *UPDhmm* uses a default minimum event size of 500 kb for both data types, which was determined empirically during simulation benchmarking.

Additional metrics

As methods can assign multiple UPD types to the same UPD event, detected UPD events were categorized into three groups right matches (correct chromosome, parental origin, and UPD type), wrong matches (chromosome and parental origin but misclassified UPD type), and uncertain matches (correct chromosome and parental origin but returned both UPD types).

Additionally, we evaluated *UPDhmm*'s accuracy in identifying the coordinates of simulated UPD events by analyzing the overlap between detected events and the actual simulated regions.

Pre-processing Steps on 1000 genomes trio data

For 1000 genomes exome data, we eliminated variants that fell within the following criteria: (1) Centromeric and pericentromeric regions, extending 2 MB upstream and downstream of each centromere, based on the UCSC genome track for the GRCh37 genome version (http://hgdownload.cse.ucsc.edu/goldenPath/hg19/database/cytoBand.txt.gz); (2) Segmental duplications as identified in the UCSC genome track (https://hgdownload.soe.ucsc.edu/goldenPath/hg19/database/genomicSuperDups.txt.gz) along with HLA and KIR regions (chr6:28,477,797-33,448,354 and chr19: 55,228,188-55,383,188, respectively); (3) Variants that were homozygous for the reference allele in the proband, mother, and father; and (4) Regions annotated as large deletions that appear in any of the individuals that constitute the trio according to the 1000 project README annotation (https://ftp.1000genomes.ebi.ac.uk/vol1/ftp/release/20110521/README.phase1_integrated_release_version3_20120430). For 1000 Genomes genome data, we eliminated variants based on the following criteria: (1) Centromeric and pericentromeric regions, extending 2 MB upstream and downstream of each centromere, using the UCSC genome track for the GRCh38 genome version (https://hgdownload.soe.ucsc.edu/goldenPath/hg38/database/centromeres.txt.gz); (2) Regions annotated as segmental duplications, according to the UCSC genome track (https://hgdownload.soe.ucsc.edu/goldenPath/hg38/database/genomicSuperDups.txt.gz) along with HLA and KIR regions (chr6:28,510,120-33,480,577 and chr19:54,025,634-

55,084,318, respectively); (3) Variants that were homozygous for the reference allele in the proband, mother, and father, consistent with the filtering of 1000 Genomes project data; and (4) Variants overlapping structural variants in any of the individuals that constitute the trio identified by the 1000 Genomes project, with the exception of inversions, which do not interfere with UPD detection (https://ftp.1000genomes.ebi.ac.uk/vol1/ftp/data_collections/1000G_2504_high_coverage/working/20220422_3202_phased_SNV_INDEL_SV/README_1kGP_phased_panel_110722.pdf)

UPDhmm evaluation in SSC cohort

Data description and pre-processing

Whole genome sequencing (WGS) data from the Simons Simplex Collection (SSC), part of the Simons Foundation Autism Research Initiative (SFARI), were used for this study. The SSC includes families in trio (proband with both biological parents) and quad (proband with an unaffected sibling and both biological parents) configurations. Variant calling was performed using the New York Genome Center's joint-calling pipeline to generate a high-quality set of single nucleotide variants (SNVs) and small insertions and deletions (indels). Before the detection of uniparental disomy (UPD) events, several filtering steps were applied. First, ten samples from five families with incorrectly matched family structures were excluded based on an excess of apparent *de novo* variants, likely reflecting sample mismatches. Second, to ensure genotype quality, only biallelic sites

with a depth of coverage (DP) greater than 15 and genotype quality (GQ) above 80 were retained. Genotypes were re-assigned based on variant allele frequency (VAF), with homozygous reference calls defined as VAF < 0.15, heterozygous defined as VAF between 0.30-0.70, and homozygous alternate calls as VAF > 0.85. Variants with intermediate VAF values (between 0.15–0.30 and 0.70–0.85) were excluded to avoid ambiguous calls. Third, variants located in centromeric and pericentromeric regions—defined as ±2 Mb surrounding each centromere—as well as those within annotated segmental duplications, and the HLA and KIR loci (chr6:28,510,120–33,480,577 and chr19:54,025,634–55,084,318, respectively), were filtered out using UCSC genome browser annotations. Additionally, variants that were homozygous for the reference allele in all trio members were excluded. Lastly, variants overlapping structural variants identified in any individual of the trio, as reported by the SFARI consortium, were also removed.

Filtering of Recurrent UPD Events

To refine the set of UPD events and minimize technical artifacts, we applied a recurrence-based filtering step. We filtered out short recurrent events with less than 100 mendelian errors, which are likely to represent technical artifacts. To do so, we first merged overlapping UPD events into genomic intervals and excluded those regions that contained events from more than two unrelated individuals. Events with more than 100 Mendelian errors were retained even if partially overlapping with smaller recurrent regions. A detailed preprocessing guide and a BED file with recurrent artefacts identified in the

SSC dataset are available in the UPDhmm Bioconductor vignette and Zenodo repository (DOI: 10.5281/zenodo.17193286)

Filtering of events with abnormal ratio depth

We calculate for every event the mean read sequencing depth between the coordinates of the detected event. Then, we defined the normalized read depth ratio as the ratio between the event's depth and the depth of the rest of the genome. We retained only those events with a normalized read depth ratio between 0.8 and 1.2 (Figure S6).

Overall filtering Strategy

Prior to final UPD call selection in the Simons Simplex Collection (SSC), several filters were applied to improve specificity, including Mendelian error count, event size, recurrence across individuals, copy number variation (CNV) status, and redundancy merging.

- **Mendelian errors:** Events with fewer than two Mendelian errors were excluded.
- **Event size:** Events smaller than 500 kb were discarded.
- **Recurrent artifacts:** UPDs observed in more than two individuals were removed, as these likely represent artifacts.
- **CNV filter:** Events showing abnormal sequencing depth compared to the sample's genome-wide average were considered likely CNVs and excluded.

- **Redundancy merging:** Events on the same chromosome and with the same UPD type were merged if they overlapped, assuming they represented a single biological event.

These filters enriched for high-confidence UPD events with potential clinical relevance while eliminating those likely caused by technical noise or confounding genomic features.

Supplementary figures

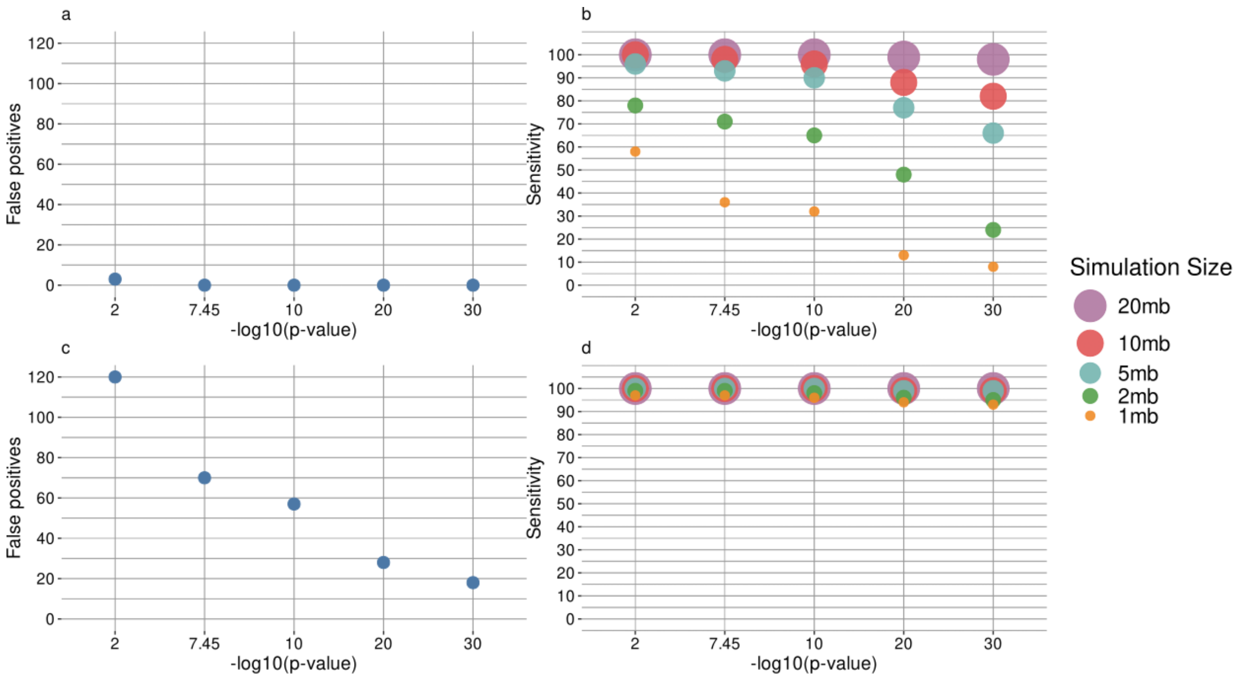

Figure *S*1. Parameters' evaluation in *UPDio*. Signals with a -log10(p-value) in *UPDio* smaller than the threshold were considered as true events. The default threshold is 7.45, as set by the original authors in the original publication. a-b: Exome data. a: False positives: events detected without simulated UPD events. b: Sensitivity across different sizes; dot size reflects the size of the simulated event. c-d: Genome data. c: False positive. d: Sensitivity

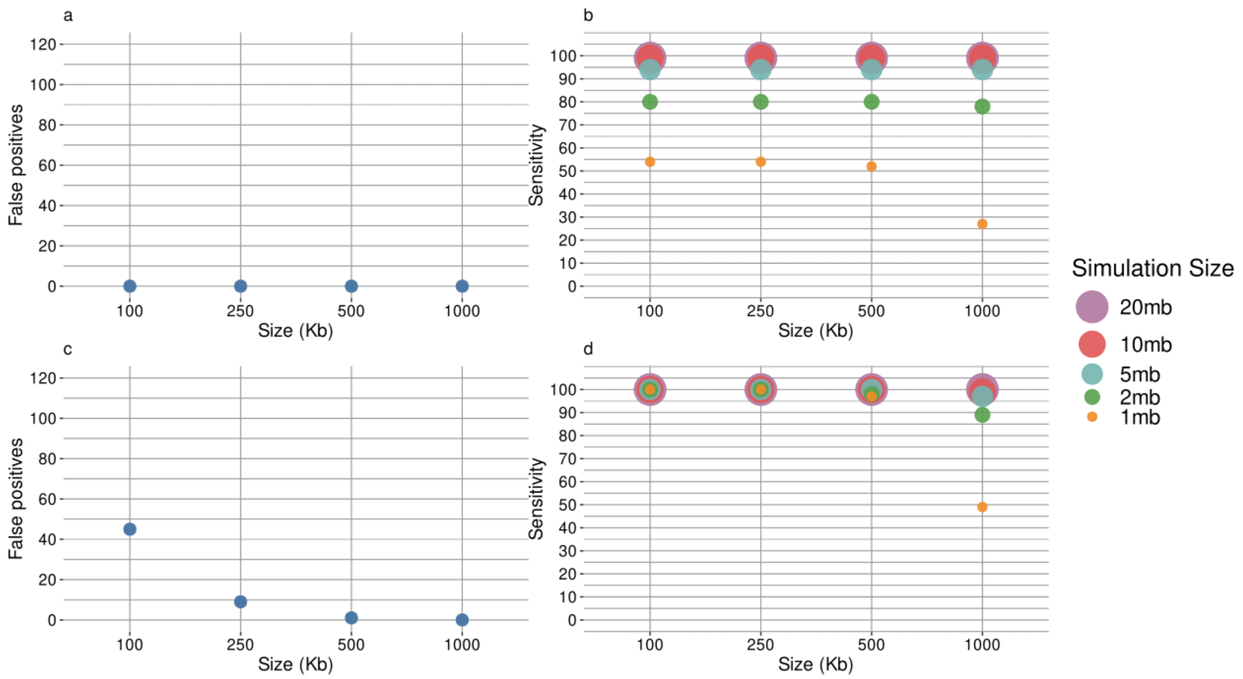

Figure S2 . Parameters' evaluation in UPDhmm. Signals larger than the threshold were considered as true events. A-B: Exome data. A: False positives: events detected without simulated UPD events. B: Sensitivity across different sizes; dot size reflects the size of the simulated event. C-D: Genome data. C: False positives. D: Sensitivity.

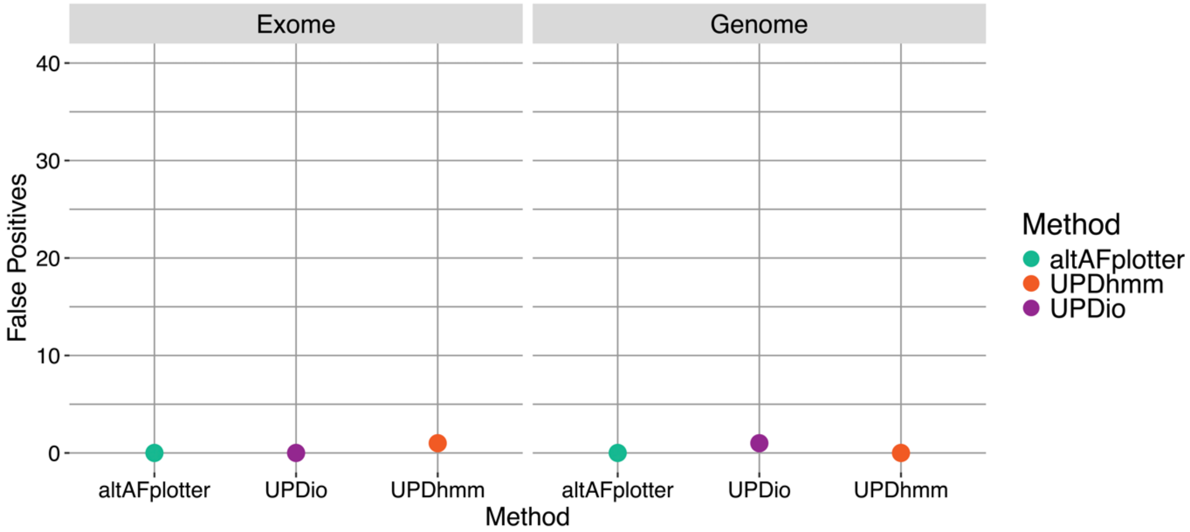

Figure S3 Figure S3. False positives after SV filtering. Comparison of false positives detected by *UPDio*, *AltAFplotter*, and *UPDhmm* after SV filtering.

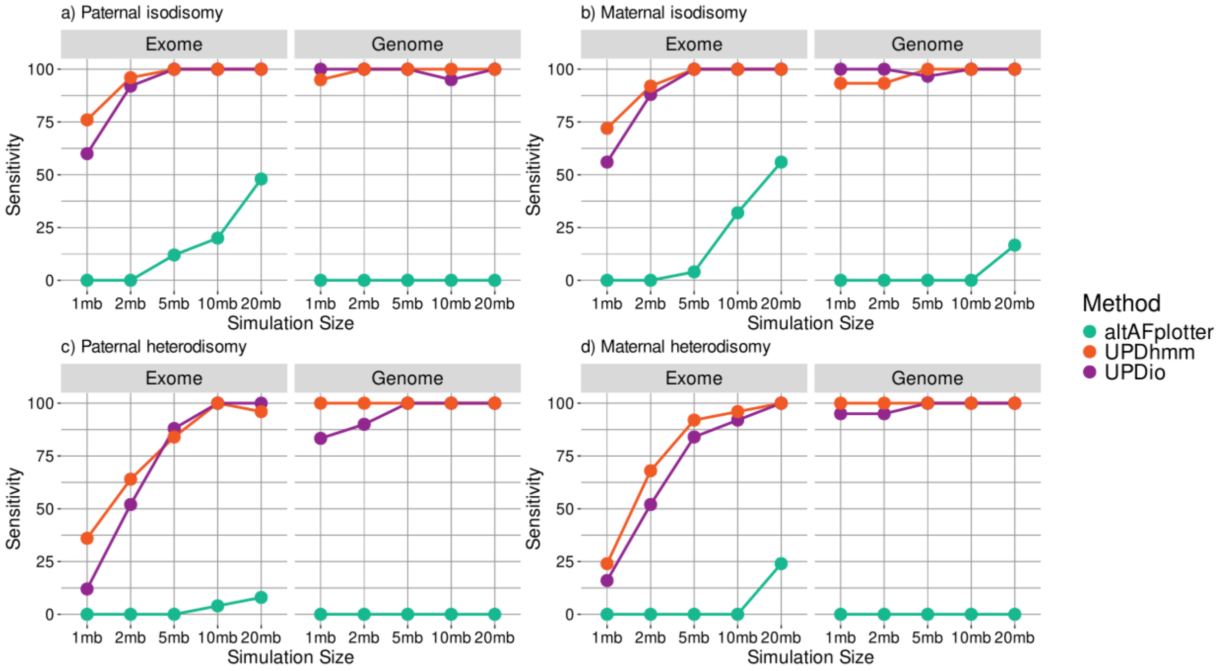

Figure S4. Sensitivity of *UPDio*, *AltAFplotter*, and *UPDhmm* across different simulated UPD types.

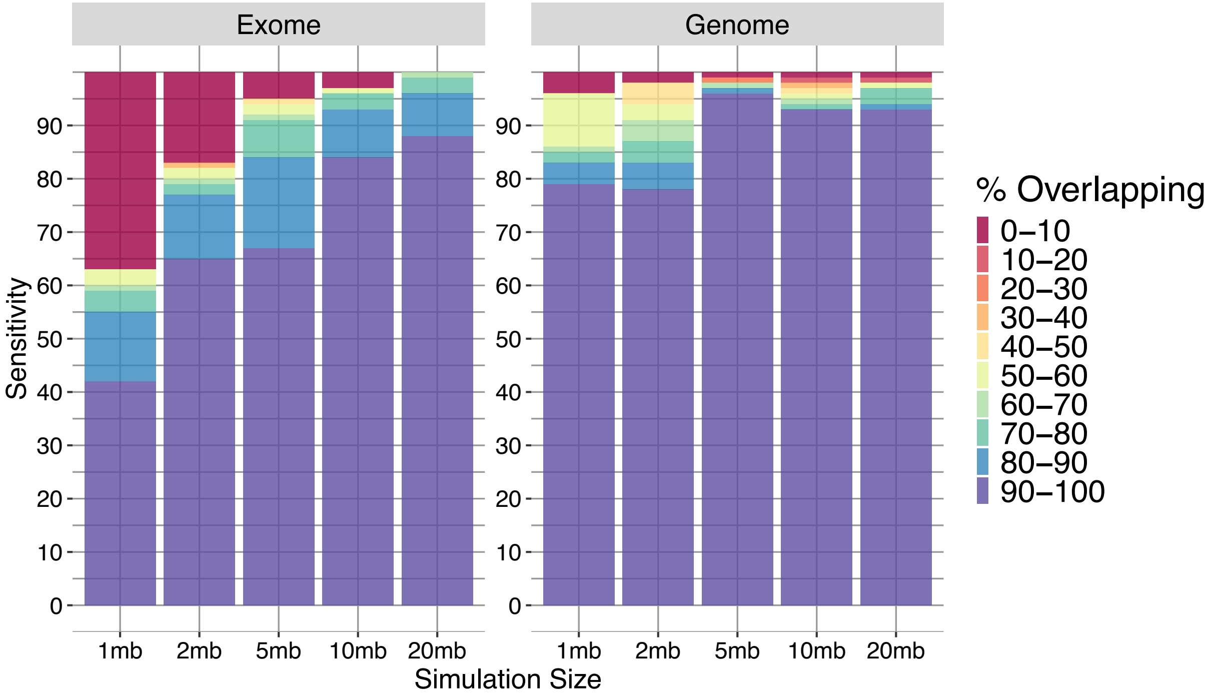

Figure S5. Percentage of overlap between simulated UPD events and those predicted by UPDhmm. This calculation presents the percentage of overlap between simulated and detected events across different simulation sizes, divided into deciles.

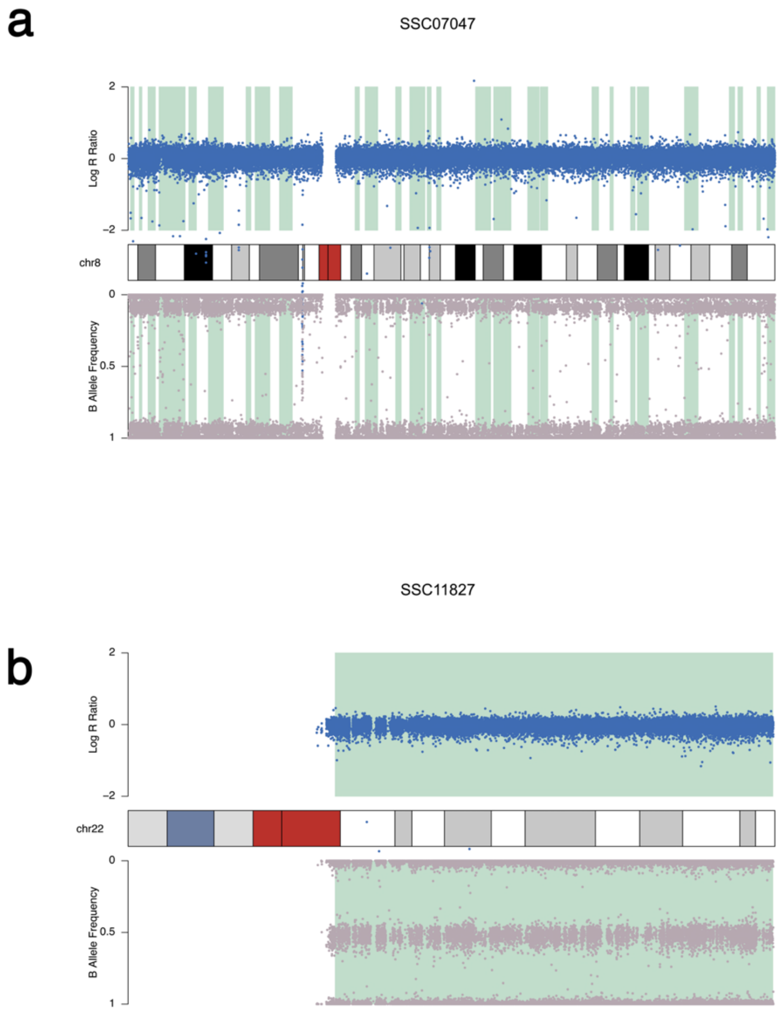

Figure S6 SNP array validation of UPD events detected by *UPDhmm*. a. Plot of SNP array data of chromosome 8 for sample SSC07047. b. Plot of SNP array data of chromosome 22 for sample SSC11827. In both figures, the upper panel shows the Log R Ratio (LRR) and the lower panel displays the B Allele Frequency (BAF) distribution, with an ideogram of the represented chromosome in between. Blue shaded regions indicate the UPD segments detected by *UPDhmm*. Positive LRR values suggest copy number gains, while negative values indicate losses. BAF plots show deviations from expected allele proportions, reflecting allelic imbalance associated with UPD.

References

King DA, Fitzgerald TW, Miller R et al. A novel method for detecting uniparental disomy from trio genotypes identifies a significant excess in children with developmental disorders. Genome Res 2014;24:673–87.

Ma X, Shao Y, Tian L et al. Analysis of error profiles in deep next-generation sequencing data. Genome Biol 2019;20:50.
